# A 6-year follow-up of a large European cohort of children with attention-deficit/hyperactivity disorder-combined subtype: outcomes in late adolescence and young adulthood

**DOI:** 10.1007/s00787-016-0820-y

**Published:** 2016-02-02

**Authors:** Marloes van Lieshout, Marjolein Luman, Jos W. R. Twisk, Hanneke van Ewijk, Annabeth P. Groenman, Andrieke J. A. M. Thissen, Stephen V. Faraone, Dirk J. Heslenfeld, Catharina A. Hartman, Pieter J. Hoekstra, Barbara Franke, Jan K. Buitelaar, Nanda N. J. Rommelse, Jaap Oosterlaan

**Affiliations:** 1 Department of Clinical Neuropsychology, VU University Amsterdam, Van der Boechorststraat 1, 1081 BT Amsterdam, The Netherlands; 2 Department of Health Sciences, VU University Amsterdam, Amsterdam, The Netherlands; 3 Department of Epidemiology and Biostatistics, VU University Medical Center, Amsterdam, The Netherlands; 4 Department of Psychiatry, Donders Institute for Brain, Cognition and Behavior, Radboud University Medical Centre, Nijmegen, The Netherlands; 5 Karakter Child and Adolescent Psychiatry University Center, Nijmegen, The Netherlands; 6 Departments of Psychiatry and of Neuroscience and Physiology, SUNY Upstate Medical University, Syracuse, USA; 7 Department of Psychiatry, University Medical Center Groningen, University of Groningen, Groningen, The Netherlands; 8 Department of Human Genetics, Radboud University Medical Centre, Nijmegen, The Netherlands; 9 Department of Cognitive Neuroscience, Donders Institute for Brain, Cognition and Behavior, Radboud University Medical Centre, Nijmegen, The Netherlands

**Keywords:** ADHD, Persistence, Symptom severity, Overall functioning, Prediction, Follow-up, Treatment

## Abstract

There are very few studies on the long-term outcome of children and adolescents with ADHD-combined type in Europe. The objective of the present study is to assess the 6-year outcome (including pharmacological treatment) of a large cohort of participants with ADHD-combined type (*N* = 347, mean age 11.4 years) in late adolescence and early adulthood. At study entry and follow-up (mean age 17.4 years), participants were comprehensively assessed on ADHD and comorbid disorders by structured psychiatric interviews and multi-informant questionnaires. Overall functioning was assessed by the Children’s Global Assessment Scale. The retention rate was 75.6 %. The majority of participants (86.5 %) persisted in a DSM-5 ADHD diagnosis, 8.4 % had a subthreshold diagnosis, and 5.1 % remitted from the disorder at follow-up. Comorbidities decreased strongly; oppositional defiant disorder: 58 > 31 %, conduct disorder: 19 > 7 %. At follow-up, mood- and anxiety disorders were virtually non-existent following strict criteria (1–3 %). Percentage of children having had pharmacological treatment at any time increased from 79 to 91 %. On the Children’s Global Assessment Scale, 48.5 % of participants were still functionally impaired at follow-up. Parental ADHD, higher ADHD symptom severity at baseline and higher parent-reported impairment at baseline positively predicted current ADHD symptom severity (*R*^2^ = 20.9 %). Younger baseline age, higher ADHD symptom severity at baseline and higher parent-reported impairment at baseline were positively associated with poorer overall functioning (*R*^2^ = 17.8 %). Pharmacological treatment had no (beneficial) impact on either ADHD symptom severity or overall functioning. Results confirm that ADHD is largely persistent into late adolescence with severity and family history for the disorder as important risk factors.

**Electronic supplementary material:**

The online version of this article (doi:10.1007/s00787-016-0820-y) contains supplementary material, which is available to authorized users.

## Introduction

Attention-deficit/hyperactivity disorder (ADHD) is characterized by symptoms of inattention and/or hyperactivity/impulsivity that lead to functional impairments in multiple domains [1]. Twenty longitudinal studies show an average childhood ADHD persistence rate of ~15 % at age 25 years, although rates in individual studies vary greatly (4–70 %) [2]. These variable findings may be explained by differences in DSM versions used to diagnose ADHD, ADHD subtypes/severity, presence of comorbid disorders, ages at study entry and follow-up, adult ADHD definitions, and whether functional impairment was taken into account. A highly consistent finding is that hyperactive/impulsive symptoms decrease over time, and inattentive symptoms remain relatively stable [3, 4]. Further, persistent ADHD is associated with major chronic problems in adult life relative to remitted ADHD, illustrated by higher rates of substance use disorders [5] and other psychiatric comorbidities [6].

As symptoms of ADHD may persist in adulthood and are related to worse outcomes in adult life, it is of great clinical relevance to investigate predictors of the course of ADHD. Retrospective studies in large representative population samples report that more family adversities [7], greater ADHD severity [7, 8], presence of comorbidities [7], and, counter-intuitively, more treatment [7, 8] in childhood predict ADHD persistence, with good accuracy (receiver operating characteristic = 0.76 [7]). Longitudinal studies confirm the predictive value of family adversities [9, 10] and comorbidities [9], and provide some support for the predictive value of ADHD familiality [9, 11]. As pharmacological treatment is currently the preferred treatment in ADHD, a very important question is whether continued pharmacological treatment is related to outcomes over time as well.

It is important to note that above-mentioned studies were limited in several ways. Most studies investigated the prediction of ADHD persistence using dichotomous outcomes (diagnosis yes/no) [7–9], as opposed to ADHD symptom severity measures. Such a continuous measure may provide a more fine-grained picture of the disorder, and may be less prone to measurement errors than categorical subtypes of ADHD [12, 13]. Second, ADHD symptom severity might not convey the whole picture. An overall measure of functioning based on social, psychological, and academic functioning more often directly relate to the wellbeing of participants. It may thus be highly relevant to investigate overall functioning as an outcome measure and so far, longitudinal studies assessing the prediction of overall functioning are scarce (but see [14]). Further, none of the longitudinal studies considered a full set of predictors together, which is important as predictors may overlap, having consequences for measures of prediction accuracy (e.g. explained variance). In addition, analyses so far yielded very limited sample sizes of remitters (*n* = 9) [9]. Another important caveat is that not many studies took pharmacological treatment into account, which may have a significant impact on ADHD outcomes [15]. Finally, there are very few longitudinal ADHD studies in samples outside the United States. Therefore, in the current study we were able to overcome these issues by predicting ADHD outcomes continuously, assessing both symptom severity and overall functioning in a large European cohort, assessing predictors together, and investigating the impact of continued pharmacological treatment.

An important aspect in studies looking at predictors for ADHD outcome is age. As significant developmental processes (neurobiological, psychological, neurocognitive) of an individual are ongoing from childhood through adolescence into adulthood, with a marked transition period in adolescence [16], predictors of ADHD outcome may be moderated by age. For example, higher ADHD severity may have a larger impact on adolescents compared to younger children, as the environment may be more demanding (e.g. school, jobs). So far, studies have investigated either a narrow age range, or did not specifically investigate effects of age. In the current study, we aimed to investigate children in the full range of childhood and adolescence, with careful consideration of linear and quadratic age-dependent effects.

The main aim of the present 6-year follow-up of a large cohort of participants with ADHD-combined type was (1) to investigate outcome, i.e. ADHD persistence rates, comorbidity rates, symptom severity and overall functioning, considering age effects, (2) to investigate baseline predictors of both ADHD symptom severity and overall functioning including demographics, ADHD familiality, ADHD severity, comorbidities and pharmacological treatment, considering age effects, and (3) to investigate the impact of continued pharmacological treatment until follow-up within the prediction models of ADHD symptom severity and overall functioning.

## Methods

### Participants

A sample of 347 participants with ADHD-combined type (ADHD/C) aged 5–19 years participated in this study. This sample was based on the Dutch part of the International Multicenter ADHD Genetics (IMAGE) study. Between 2003 and 2006, the IMAGE study recruited families with at least one child with clinically diagnosed ADHD/C and at least one additional sibling regardless of possible ADHD status. Clinical diagnosis of each participant was assessed by health-care professionals from clinical child care centers in The Netherlands. In addition, the diagnosis was confirmed, using an extensive assessment protocol described below. The original sample (*N* = 459) was contacted and invited for follow-up on average 6.0 years (SD = 0.7) after enrolment; 75.6 % (*N* = 347) was retained successfully.

Selection procedures have been detailed previously [17]. Briefly, inclusion criteria for the IMAGE study were an age of 5–19 years, Caucasian descent, IQ ≥70, no diagnosis of autism, epilepsy, general learning difficulties, brain disorders and known genetic disorders. Parent and teacher questionnaires were used to screen participants: Conners’ long version [18] and Strengths and Difficulties Questionnaire (SDQ [19]). *T* scores ≥63 on the Conners DSM-IV ADHD subscales Inattention (*L*), Hyperactivity/impulsivity (*M*), and Total symptoms (*N*), and scores ≥90th percentile on the SDQ Hyperactivity subscale were considered clinical. Participants scoring clinically on any of these subscales were administered the Parental Account of Children’s Symptoms (PACS), a semi-structured, standardized, investigator-based interview with the parents as informants [20]. See [21] for the algorithm used to derive each of the 18 ADHD symptoms as defined by Diagnostic and Statistical Manual of mental disorders: (DSM-IV-TR [1]). Only participants with a diagnosis of ADHD/C based on the algorithm at baseline were included in the current study. Participants came from 282 different families, 81.6 % was male. Mean age at baseline was 11.4 years (SD = 2.8) and mean age at follow-up was 17.4 (SD = 2.8).

### Diagnostic, symptom severity and overall functioning assessment

At follow-up, participants were screened for ADHD using the Schedule for Affective Disorders and Schizophrenia for school-age children—present and lifetime version (K-SADS), a semi-structured, standardized, investigator-based interview with the parents as informants, and when children were 12 years or older, also with the child (separately) [22]. Participants with elevated scores on any of the screen items were administered the full ADHD interview. Additionally, parents completed the Conners’ Parent Rating Scale-Revised: Long version (CPRS-R:L [18]) and the Conners’ Teacher Rating Scale-Revised: Long version (CTRS-R:L [23], applied for participants <18 years), or the Conners’ Adult ADHD Rating Scales-Self-Report: Long Version (CAARS-S:L [24], applied for participants ≥18 years). A diagnostic algorithm was used to establish ADHD status according to DSM-5 criteria, which was similar to the algorithm used at baseline (for full description of diagnostic procedures see [25]). ADHD subtypes (combined, inattentive, or hyperactive/impulsive subtype) were established following DSM-5 criteria [26]. Comorbidities were assessed using the PACS at baseline and using the K-SADS at follow-up. Classifications in both interviews were established according DSM-IV criteria for oppositional defiant disorder (ODD) and conduct disorder (CD). Classifications of DSM-IV anxiety-, mood-, and tic disorders were established in the K-SADS at follow-up.

For both the K-SADS and the PACS, interviewers underwent comprehensive training by a team under the supervision of E. Taylor at the London Institute of Psychiatry (IoP; PACS) or JB at the Donders Institute for Brain, Cognition and Behavior, Radboud University Medical Centre, Nijmegen (K-SADS). If additional interviewers were used, each center was responsible for their training and supervision. Inter-rater agreement for the PACS was 0.88 (range 0.71–1.00) and for the K-SADS 0.94 (ADHD), 0.89 (ODD), and 0.95 (CD) [17, 25]. The interviewers were trained clinicians (child psychiatrists, psychologists) or trained researchers. Persistence of ADHD was defined as meeting full DSM-5 criteria of ADHD/C at baseline, and meeting full DSM-5 criteria of ADHD regardless of subtype, at follow-up. Subthreshold persistence of ADHD was defined as meeting full criteria of ADHD/C at baseline, and meeting criteria of subthreshold ADHD at follow-up: <6 symptoms of inattention and hyperactivity/impulsivity, but ≥4 symptoms of inattention and/or hyperactivity/impulsivity at follow-up for children <18 years. For participants ≥18 years, thresholds were five and three symptoms respectively. Remission of ADHD was defined as meeting full criteria of ADHD/C at baseline, and not meeting criteria of (subthreshold) ADHD, any subtype, at follow-up.

To assess dimensional persistence, symptom change scores were calculated by subtracting follow-up raw scores from baseline raw scores on the CPRS-R:L ADHD scales *L* (Inattention), *M* (Hyperactivity/impulsivity), and *N* (Total symptoms) [18]. Current ADHD symptom severity, and inattentive and hyperactive/impulsive symptom severity were assessed with the follow-up raw scores on the CPRS-R:L scales *N*, *L* and *M*, respectively. Scores on the Conners’ ADHD subscales represent combined measures of the number and severity of symptoms.

To measure current overall functioning, the Global Assessment Scale-score of the K-SADS (K-GAS) was administered at follow-up. After finishing the K-SADS interview, the interviewer rated psychological, social and academic functioning, resulting in an overall measure of the current level of functioning ranging between 1 (worst possible level of functioning) and 9 (best possible level of functioning) [27].

### Predictors and covariates

All predictor variables were assessed at baseline. Five different classes (italic) were investigated. *Demographic variables*: age, sex, and socio-economic status (SES) were measured. SES was calculated from the average educational levels of the parent(s), with educational levels ranging between 0 (no education) and 11 (university), according to an adapted Hollingshead scale [28] fitting the Dutch educational system. *ADHD familiality*: ADHD familiality was investigated by measuring the percentage of siblings with ADHD according to the PACS interview [20], and by establishing current parental ADHD status, based on the K-SADS interview (none versus one/both parent(s) with ADHD). *ADHD characteristics*: ADHD characteristics included ADHD symptom severity, impairment and age of ADHD onset. Symptom severity was measured by the raw score on scale *N* (range 0–54) of the CPRS-R:L [18]. Impairment was measured using both the parent SDQ [19] and teacher SDQ (range 0–15) (parent and teacher ratings correlated *r* = 0.18 and were not combined). Age of onset of ADHD was assessed using the PACS interview [20]. *Comorbidities*: Comorbid DSM-IV defined ODD (yes/no), CD (yes/no) and a screening of the presence of mood/anxiety symptoms (yes/no) were assessed with the PACS [29]. *Pharmacological treatment*: cumulative intake of psychostimulants from age of onset until our baseline measurement and from age of onset until follow-up were calculated. Lifetime medication transcripts from pharmacies were available for 87 % and covered the lifespan for 31 % of participants. In addition, a questionnaire was administered to all participants and parents, which assessed lifetime history of psychostimulant medication. When pharmacy transcripts did not fully cover the self-reported treatment period, medication parameters of the missing period(s) were calculated from the questionnaire data and were added to the measures derived from the pharmacy. To optimally take into account daily dose and duration of pharmacological treatment, cumulative intake was calculated by multiplying the mean daily dose (average dose in milligrams for all exposed days; in line with prescription guidelines [30] and given larger direct effects of dexamphetamine on dopaminergic neurotransmission [31], dexamphetamine dose was multiplied by 2) with treatment duration corrected for age (treatment duration in months divided by [age minus the minimum start-age within the sample, i.e., 28 months]; see for further details [32]).

Two potential covariates were investigated. If the univariate relationship between follow-up interval and outcome measures was significant, follow-up interval was entered as a covariate in all subsequent analyses. In addition, study site was entered as a covariate in the final prediction models.

### Procedure

At baseline, families were recruited from clinics and via advertisements. Testing took place at the VU University Amsterdam or at the Donders Institute in Nijmegen. Participants were 48 h off medication during both baseline and follow-up assessments. All ratings of behavioral functioning pertained the participant’s functioning off medication. Families were financially compensated for participating in the study. Informed consent was signed by all participants at both measurements, and parents signed for all children in their family as well. The study was approved by the national ethics committee.

### Statistical analysis

The percentage of missing data was <5 % for ADHD diagnoses, current ADHD symptom severity and overall functioning measures, 19 % for parental ADHD status, and between 0 and 9 % for the other predictors. Missing value analysis (expectation maximization) was performed for participants with one or two missing items on CPRS-R:L subscales, using all data reported in this study (scale *L*: 9 participants, scale *M*: 18 participants). K-GAS-scores were normalised by applying a Van der Waerden transformation.

For our first research question, ADHD persistence rates, percentage of comorbidities, mean symptom change and overall functioning scores were calculated. It was tested whether symptom severity decreased significantly over time and whether changes in hyperactivity/impulsivity symptom severity over time differed from changes in inattention symptom severity. To optimally correct for the familial dependency in our data, Generalized Estimating Equation analyses (GEE) were used, with an exchangeable correlation structure. Additionally, interaction-effects of symptom change between baseline and follow-up with age/age^2^ were tested. For our second research question, an optimal set of predictors for current ADHD symptom severity and overall functioning in participants with ADHD/C was derived in three successive analysis steps. In step 1, GEE analyses were ran on each of the five classes of predictors separately (see Supplemental Table S1 and Supplemental Table S2, available online), with current ADHD symptom severity or overall functioning as outcome measure respectively. The mean correlation between all predictors was 0.09 (0.001 < *r* < 0.45), indicating no collinearity. For all predictors, we tested linear effects with outcome measures, except for age. Literature indicated a possible non-linear (quadratic) relationship for the relation between age and our outcome measures [3]. In step 2, predictors with a *p* value <0.15 in step 1 were entered into the final GEE model. Finally, in step 3, a backward selection (variables deleted when *p* >0.05) procedure was performed for model optimization. Additionally, to investigate possible moderating effects of age on the models for ADHD symptom severity and overall functioning, interactions between both age (assessed at baseline) and age^2^ and significant predictors of outcome were added to the final model. When an interaction-effect with age or age^2^ was significant, the finding was further explored by testing the final model in subsamples subdivided based on age at baseline (<12 years and ≥12 years). Final models were further tested separately for symptom severity of inattention and separately for hyperactivity/impulsivity as outcome measures, to explore whether the model was applicable to both symptom dimensions. Further, as the reliability and validity of the CPRS-R:L is only established for children under 18 years of age, we tested whether results of the final model for current ADHD symptom severity replicated in a subsample of children younger than 18 years. Second, the effect of missing data on the final models for both outcome measures was investigated, testing the final model using only participants with complete data. For our third research question, the first three steps of our GEE model were repeated, except that pharmacological treatment until baseline was replaced by pharmacological treatment until follow-up.

## Results

### Attrition analysis

Attrition was investigated by comparing participants successfully followed up (75.6 %) with participants lost to follow-up on variables reported in this study available at baseline. No significant group differences were found (0.13 < *p* < 0.95).

### Current ADHD-related outcomes

Table 1 shows the descriptives at baseline and follow-up of ADHD diagnosis, comorbidities, symptom severity and overall functioning. Supplemental Table 3 shows these characteristics in younger and older children (<12 years and ≥12 years). Of 459 children with ADHD/C at baseline, 333 children (72.5 %) had available information on categorical diagnoses at follow-up; 86.5 % of them persisted in a full ADHD diagnosis [51.4 % ADHD/C, 39.6 % ADHD inattentive-type (ADHD/I), 9.0 % ADHD hyperactive/impulsive-type (ADHD/H)], 8.4 % had a subthreshold diagnosis, and 5.1 % remitted from the disorder. In younger and older children these rates were comparable (*χ*^2^ = 2.871, *p* = 0.720). Regarding comorbidities at baseline, ODD was apparent in 58.0 % of the participants, CD in 18.9 % of the participants. At follow-up, ODD was apparent in 30.8 % of the participants, CD in 6.6 %, tic disorders (any type) in 2.1 %, mood disorders (depression or dysthymia) in 1.8 %, and anxiety disorders in 2.5 % of the participants. Regarding pharmacological treatment, at baseline, 78.8 % of the participants have had pharmacological treatment for their ADHD symptoms at any time, compared to 90.9 % of the participants at follow-up. At follow-up, 87.1 % of participants used methylphenidate (immediate release) at any time, 65.4 % used methylphenidate (extended release), and 6.5 % of participants used dexamphetamine.

**Table 1:** Characteristics of children with ADHD/C at baseline and follow-up

	Mean	SD
Baseline		
Demographic variables		
Age (years)	11.41	2.78
Sex *(N*/% male)	283	81.6
SES (average educational level of the parents)	5.39	2.21
ADHD familiality		
ADHD status siblings (% of siblings with ADHD)	63.55	26.01
Parental ADHD status (*N*/% ADHD in one or both parents)	96	34.2
ADHD severity^a^		
CPRS-R:L total symptom severity (scale *N*)	35.51	8.57
CPRS-R:L inattentive symptom severity (scale *L*)	18.59	4.92
CPRS-R:L hyperactive/impulsive symptom severity (scale *M*)	16.92	5.19
SDQ Impairment		
Parent	12.37	3.91
Teacher	8.03	3.17
Age of onset first ADHD symptoms (years)	2.25	1.52
ADHD pharmacological treatment		
Mean daily dose (milligram, unit equivalents)	13.31	12.92
Cumulative intake of psychostimulants	53.20	73.74
Comorbidities		
PACS ODD diagnosis (yes)	184	58.0
PACS CD diagnosis (yes)	60	18.9
PACS screen anxiety/depression (yes)	188	59.3
Follow-up		
Demographic variables		
Age at follow-up (years*)*	17.36	2.79
ADHD severity^a^		
CPRS-R:L total symptom severity (scale *N*)	23.27	11.38
CPRS-R:L total symptom severity change score (scale *N*)	12.24	11.69
CPRS-R:L inattentive symptom severity (scale *L*)	13.85	6.55
CPRS-R:L inattentive symptom severity change score (scale *L*)	4.74	6.79
CPRS-R:L hyperactive/impulsive symptom severity (scale *M*)	9.42	5.98
CPRS-R:L hyperactive/impulsive symptom severity change score (scale *M*)	7.50	6.28
ADHD pharmacological treatment		
Mean daily dose (milligram, unit equivalents)	22.04	15.74
Cumulative intake of psychostimulants	126.00	120.49
Status at follow-up		
Kiddie-Global Assessment Scale at follow-up	6.42	1.14
ADHD persistence (*N*/%)	288	86.5
ADHD/C (*N*/%)	148	51.4
ADHD/I (*N*/%)	114	40.6
ADHD/H (*N*/%)	26	9.0
Subthreshold ADHD (*N*/%)	28	8.4
ADHD remitter (*N*/%)	17	5.1
Comorbidities at follow-up		
ODD (*N*/%)	103	30.8
CD (*N*/%)	22	6.6
Tic disorder (*N*/%)	7	2.1
Mood disorder (*N*/%)	6	1.8
Anxiety disorder (*N*/%)	8	2.5

*ADHD* attention-deficit/hyperactivity disorder, *ADHD*/*C* attention-deficit/hyperactivity disorder-combined type, *ADHD*/*H* attention-deficit/hyperactivity disorder-hyperactive/impulsive type, *ADHD*/*I* attention-deficit/hyperactivity disorder-inattentive type, *CD* conduct disorder, *CPRS-R:L* Conners’ parent rating scale-revised: Long version *ODD* oppositional defiant disorder, *PACS* parental account of children’s symptoms, *SDQ* strengths and difficulties questionnaire, *SES* socio-economic status

^a^Combined measures of parent/self and teacher report

Total raw ADHD symptom severity on the CPRS-R:L scale *N* decreased from 35.51 to 23.27 (*p* < 0.001, mean change = 12.24, SD = 11.69). Inattentive raw symptom severity on the CPRS-R:L scale *L* decreased from 18.59 to 13.85 (*p* < 0.001, mean change = 4.74, SD = 6.79), and hyperactive/impulsive raw symptom severity on the CPRS-R:L scale *M* decreased from 16.92 to 9.42 (*p* < 0.001, mean change = 7.50, SD = 6.28). The decrease in hyperactivity/impulsivity was larger than the decrease in inattention (*p* < 0.001). Interaction effects of symptom change between baseline and follow-up with age/age^2^ were significant for inattention (*b* = 0.42/0.02 and *p* = 0.003/0.004, respectively), showing that inattentive symptoms decreased more in older than younger children, then leveling off around the age of 16–18 (age at follow-up).

Of 332 participants with current K-GAS-scores available, 161 participants (48.5 %) were functionally impaired at follow-up (K-GAS-score ≤6). Eight participants (2.4 %) had optimal functioning scores (K-GAS-score = 9). Of 288 participants with persistent ADHD and current K-GAS scores available, 153 participants (53.1 %) were functionally impaired at follow-up. Two participants (0.7 %) with persistent ADHD had optimal functioning scores. Older age was associated with better overall functioning, as reflected in a higher current K-GAS-score (*b* = 0.09, *p* < 0.001).

### Prediction of current ADHD symptom severity and overall functioning

Given our high ADHD persistence rates, prediction models were tested only for our dimensional measures of ADHD. Table 2 shows the final prediction models for current ADHD symptom severity. Higher current ADHD symptom severity was predicted by positive parental ADHD status, higher baseline ADHD symptom severity, and higher parent-reported baseline impairment, explaining 20.9 % of variance.

**Table 2:** Final prediction model for current ADHD symptom severity in children with ADHD/C

	*B*	*b* ^a^	SE	*p*
Parental ADHD status	0.15	3.53	1.24	0.004
CPRS-R:L symptom severity	0.26	0.35	0.08	<0.001
SDQ parent-reported impairment	0.25	0.74	0.19	0.003
		*R* ^2^ = 20.89 %		

*ADHD* attention-deficit/hyperactivity disorder, *CPRS-R:L* Conners’ Parent Rating Scale-Revised: long version *SDQ* strengths and difficulties questionnaire

^a^Unstandardized regression coefficient

Table 3 shows the final prediction model for current overall functioning. Lower K-GAS-scores were predicted by younger age at baseline, higher baseline ADHD symptom severity, and higher parent-reported baseline impairment, explaining 17.8 % of variance.

**Table 3:** Final prediction model for the current global assessment scale in children with ADHD/C

	*B*	*b* ^a^	SE	*p* ^b^
Age at baseline	0.15	0.05	0.02	0.008
CPRS-R:L symptom severity	−0.26	−0.02	0.004	<0.001
SDQ parent-reported impairment	−0.15	−0.04	0.01	0.004
		*R* ^2^ = 17.75 %		

*ADHD* attention-deficit/hyperactivity disorder, *CPRS-R:L* Conners’ Parent Rating Scale-Revised: long version *SDQ* strengths and difficulties questionnaire

^a^Unstandardized regression coefficient

^b^Models are corrected for follow-up interval

For current ADHD symptom severity none of the predictors interacted significantly with age or age^2^ (0.12 < *p* < 0.58). For current overall functioning, there was a significant interaction between age and parent-reported impairment (*p* = 0.029). Further exploration showed that parent-reported impairment was a significant predictor of current overall functioning only in the subsample of younger participants (*p* < 0.001) and not in the subsample of older participants (*p* = 0.64). The prediction model for current ADHD symptom severity (including parental status of ADHD, baseline symptom severity, and baseline parent-reported impairment) remained significant (0.000 < *p* < 0.027) when tested for ADHD inattention symptom severity (*R*^2^ = 14.7 %) and ADHD hyperactivity/impulsivity symptom severity (*R*^2^ = 20.2 %).

All predictors in the final model for current ADHD symptom severity remained significant with similar relationships when tested in a subsample with children younger than 18 years (0.002 < *p* < 0.010, *R*^2^ = 16.9 %), demonstrating that the findings were not the result of applying the CPRS-R:L in children of 18–24 years old. Findings for current ADHD symptom severity replicated when cases without complete data were removed from the analyses (0.001 < *p* < 0.012, *R*^2^ = 21.4 %). Predictors in the final model for current overall functioning remained significant (baseline symptom severity) or marginally significant (age, baseline parent-reported impairment; 0.001 < *p* < 0.069, *R*^2^ = 16.8 %).

### Continued pharmacological treatment

To investigate whether continued pharmacological treatment is related to better outcomes (hypothesis 3), pharmacological treatment until follow-up was added to the model predicting current ADHD symptom severity and overall functioning. In the first model, one additional predictor was now significant; more continued pharmacological treatment also predicted higher symptom severity (*b* = 0.011, *p* = 0.020). The total model explained 22.5 % of variance. For overall functioning, continued pharmacological treatment until follow-up did not contribute to the model.

### Covariates

Follow-up interval was significantly related to the K-GAS-score (*b* = −0.25, *p* = 0.001), but not to ADHD symptom severity (*b* = 1.64, *p* = 0.22), and therefore was added to the model of overall functioning as a covariate for all subsequent analyses. Further, study site was a non-significant predictor in both the final model of ADHD symptom severity (*b* = 0.22, *p* = 0.87) and of overall functioning (*b* = 0.10, *p* = 0.31). Adding this covariate to these final models did neither change direction of effects of predictors, nor their significance (*p* < 0.05).

## Discussion

The current European prospective study investigated the course of ADHD/C from childhood into late adolescence/young adulthood and studied a full set of potential important predictors for ADHD outcomes: current symptom severity and overall functioning. Importantly, the additional value of continued pharmacological treatment was examined. In summary, although symptom severity decreased, persistence rates were indisputably high: the vast majority of participants had a persistent DSM-5 ADHD diagnosis (86.5 %), independent of age. The greater part of ADHD persisters still met combined type criteria (51.4 %). About half (48.5 %) of participants were still functionally impaired at follow-up [22]. At follow-up, comorbidity rates (ODD, CD) decreased strongly compared with the baseline measurement. Mood and anxiety disorders were virtually non-existent following strict criteria (1–3 %). The large majority of participants (>90 %) had taken stimulants at some point in time. Predictive variables together explained up to 20 % of variance in our outcome measures: higher ADHD symptom severity and higher parent-reported impairment predicted higher current ADHD symptom severity and lower overall functioning. Positive parental ADHD status contributed to the prediction of higher current ADHD symptom severity, while being younger at initial participation predicted lower overall functioning. Age further was important as a moderator in the prediction of overall functioning: parent-reported impairment was predictive only in younger children (<12 years). Continued pharmacological treatment was of no relevance for overall functioning at follow-up, and against our expectation, pharmacological treatment was positively related to symptom severity at follow-up.

As noted above, ADHD symptom severity decreased significantly, but symptom decrease was only slight, and many adolescents and young adults still fulfilled criteria for a DSM-classification. Although this prospect was expected for the younger part of our sample, a striking finding is that persistence rates were similarly high in the older part of the sample (age up to 25). An important explanation may be our stringent inclusion criteria applied at baseline: ADHD-combined criteria had to be met by all participants, a subtype that was found highly predictive of a persistent course [7]. Adolescents still meeting full criteria for ADHD/C may be considered as relatively more severely affected compared to younger children meeting these criteria. This may have resulted in one of the highest reported persistence rates thus far; even higher than the 70 % persistence rate that was found in the Langley study [10]. The rate of mood and anxiety disorders at follow-up in our sample is lower compared to most other studies [33]. It is possible that participants with higher rates of mood or anxiety disorders were less willing to participate at follow-up. However, there were no differences in comorbidity measures at baseline between participants successfully followed-up or participants lost to follow-up, making this suggestion less likely. Possibly, comorbid problems in ADHD emerge early in childhood and may remit during adolescence, which is what our findings regarding the decrease of ODD and CD rates suggest. For mood disorders, this idea was supported by a study of Biederman and colleagues, showing that comorbid rates of mood disorders in participants with ADHD in adulthood seem comparable to those in a control group, regardless of higher levels of mood disorders earlier in life in participants with ADHD [34]. For both mood and anxiety disorders, another explanation may (also) hold true. Comparing our results with similar studies that used strict DSM-IV criteria with both parents and participants as informants, an important difference is that we considered a disorder present when it was apparent at the time of assessment, while other studies used wider time-intervals, e.g. considering a disorder present when apparent within the past 3–5 years [9, 34].

Our study clearly confirms that ADHD/C is a strongly pervasive disorder at least until adolescence/young adulthood. The relative stability of ADHD symptoms is especially important given that the adolescent brain develops strongly during the transition from puberty into adulthood, marked by increased reward seeking activities leading to problematic decision-making processes [16]. In combination with ongoing symptoms of ADHD, this may have unfavorable effects on academic, health, and social outcomes, and may lead to adverse outcomes such as offending behavior. On the other hand, offering a more positive perspective, comorbidity rates decreased strongly and about half of our sample was not severely impaired, but had a moderate level of overall functioning.

Current symptoms and overall functioning were predicted by a few variables only, which together explained up to 20 % of variance. Not surprisingly, having many symptoms and being highly impaired at baseline predict worse future outcomes, which was also reported by other [9, 35], but not all, studies [11]. The positive predictive value of parental ADHD for current symptom severity is consistent with studies showing that broader concepts as ‘family history of ADHD’ or ‘parental psychopathology’ are related to ADHD outcomes [9, 10]. This finding may be attributed to genetic factors, but it is also possible that this predictive effect relates to family-environmental factors. As we have shown, parental status of ADHD is a more important predictor than having siblings with ADHD. Given that siblings and parents share a comparable genetic make-up with the proband, and siblings usually have less influence on upbringing than their parents, our finding may indicate an important role for family-environmental factors in the course of ADHD. Finally, younger age at baseline predicted lower overall functioning as well as higher ADHD severity, but the latter only in combination with continued pharmacological treatment. An explanation for this finding is that the world is expanding more for older children compared to younger children. The impact of symptom severity may be relatively smaller then, or may add positively to overall functioning as risk taking behaviour may increase, which can be interesting for peers on that age.

Interestingly, our prediction models for ADHD outcomes were largely independent of the developmental phase of participants, confirming the stability of our predictors in a sample of children with ADHD/C with a large age range. Only for current overall functioning parent-reported impairment was a significant predictor in younger children, which may be explained by the smaller involvement of parents when children grow older, with a decrease in correct judgement of impairment levels of their child accordingly.

Cumulative intake of psychostimulant medication neither at baseline nor at follow-up had a beneficial impact on ADHD outcomes or overall functioning in our sample. Moreover, higher cumulative intake at follow-up predicted worse outcomes in terms of ADHD severity. Although ADHD symptoms have been shown to decrease with stimulant treatment, our findings do not support long-term positive effects on outcome. Three other studies also found that ADHD treatment (any type) positively predicted ADHD persistence [8, 36], or ADHD severity [10], which was interpreted as treatment being a proxy of ADHD severity. This explanation may also underlie our current finding. In line with this, the results of the Multimodal Treatment study of ADHD (MTA), a large randomized controlled trial comparing the effects of systematic pharmacological treatment, behavioral therapy, a combination of these two, or usual community care, suggested that initial benefits of pharmacological treatment on cognitive functioning and symptom severity dissipated from 2 years on [35]. Accordingly, a recent study showed that when psychostimulant medication intake increased in a population of children with ADHD, there were no positive effects on functional outcome measures such as academic outcomes or schooling attainment [37]. Conversely, evidence for negative effects on mood and non-serious adverse events (e.g. sleeping problems, decreased appetite) were evident [37, 38], indicating that, in line with our findings, the long-term effects of pharmacological treatment on functional outcomes of ADHD may need reconsideration [39].

Although we were able to explain a moderate amount of variance in current symptom severity and overall functioning, many of the baseline predictor variables were unrelated to outcomes including sex, SES, age of onset, and comorbidities. As we investigated sets of predictors together, it may be that comorbidities were not included in the model as they may overlap with ADHD symptom severity measures. Indeed, ODD was related to ADHD symptom severity in the separate class analyses but did not retain in the model with other predictor variables. Two other studies showed a somewhat larger role for the predictive value of CD [9, 40]. The first study showed that comorbid CD at baseline was significantly more often seen in ADHD persisters or late remitters (after the age of 12 years) compared to participants that remitted from the disorder before the age of 12 years [9]. In the second study, childhood CD as a comorbid condition predicted failure to graduate and young parenthood compared to ADHD childhood CD [40]. The discrepancies between these two studies and our findings regarding the predictive value of comorbid CD may be explained by a variety of factors: e.g. the use of different definitions or operationalization of dependent and outcome measures, including different samples or using a different statistical or methodological approach. Further, one study showed that comorbidities were predictive of persistence when using a combined measure [7], indicating that a measure of ‘general vulnerability’ for developing persisting disorders may be of importance. For the other non-significant predictors, our findings are in line with the current literature [7–9, 11].

### Limitations and future recommendations

Although we studied a relatively large sample of participants with ADHD/C compared to other studies, the sample may have been relatively small for our analyses including interaction-effects with age. Second, including participants with other types of ADHD (such as ADHD/I) would have allowed us to investigate whether these subtypes showed higher remittance rates than ADHD/C. Further, participants were all of Caucasian origin, limiting generalisation to a broader ethnic population. Fourth, interviewers were not systematically blinded for diagnostic status when administering diagnostic interviews. However, since both families and interviewers had no specific interest in the outcome measures in our study, we don’t consider this a major bias in the results. Fifth, different diagnostic interviews were used at baseline (PACS) and at follow-up (K-SADS). As both interviews systematically investigated similar DSM criteria for ADHD, we do not expect this difference to have a major impact on our results. Finally, our study was restricted to children with a clinical diagnosis, whereas some previous studies recruited participants with ADHD symptoms from the general population [6, 41] who may be less severely affected, resulting in higher remission rates. Also, it should be of note that persistence rates may decrease more with older age in adulthood [6].

As the majority of variance in ADHD outcomes remain unexplained, far more studies are warranted for a more accurate prediction of future ADHD outcomes. For example, studies should include other variables that relate to family-environmental factors, such as upbringing style or attachment style. Perhaps multiple domains can be integrated in one model, by also including genetic variations, structural and functional brain measures, and neurocognitive factors [42–45].

## Conclusion

Our study demonstrates that combined type ADHD strongly persists into late adolescence and young adulthood, posing a challenge for adolescence and adult mental health care as problems do not disappear. Risk factors for worse outcomes in adolescence or young adulthood include high ADHD symptom severity and impairment, younger age, parental ADHD, and more continued pharmacological treatment. The developmental phase of participants was of little importance for our predictors for ADHD outcome, showing the importance of these predictors for the entire ADHD sample. In an era in which pharmacological treatment is the preferred type of intervention, our finding that pharmacological treatment had no positive predictive value for ADHD-related outcomes is clinically important and substantiates further study into this topic. Finally, it is of great relevance for future work to discover the factors that cover the 80 % unexplained variance of our models that predict future outcome, to give direction to develop newer and potentially more effective interventions.

## Electronic supplementary material

Below is the link to the electronic supplementary material.

Supplementary material 1 (DOCX 25 kb)
