# Supplementary material for: A 6-year follow-up of a large European cohort of children with attention-deficit/hyperactivity disorder-combined subtype: outcomes in late adolescence and young adulthood
Source: Eur Child Adolesc Psychiatry. 2016 Feb 2;25:1007–17. doi: 10.1007/s00787-016-0820-y (PMC4990613; doi:10.1007/s00787-016-0820-y)
Supplement: Supplementary file 1 — Supplementary material 1 (DOCX 25 kb) [file 787_2016_820_MOESM1_ESM.docx]

**Table 1 Supplement.** Pre-selection of Predictors for Current ADHD Symptom Severity

|  | *b^b^* | *SE* | *p^c^* |
| --- | --- | --- | --- |
| **Class 1: Demographic variables** |  |  |  |
| Age (*yrs*) | -0.50 | 0.23 | **.026** |
| Sex (male=0, female=1) | -3.13 | 1.77 | **.078** |
| SES (average educational level of the parents) | -0.33 | 0.28 | .238 |
| **Class 2: ADHD familiality** |  |  |  |
| ADHD status siblings (% of siblings with ADHD) | -0.03 | 0.03 | .381 |
| Parental ADHD status (ADHD in one or both parents) | 4.91 | 1.57 | **.002** |
| **Class 3: ADHD characteristics** |  |  |  |
| CPRS-R:L symptom severity (scale N) | 0.32 | 0.07 | **<.001** |
| SDQ parent-reported impairment | 0.81 | 0.17 | **<.001** |
| SDQ teacher-reported impairment | -0.31 | 0.21 | **.134** |
| Age of onset first ADHD symptoms (*yrs*) | -0.16 | 0.34 | .637 |
| **Class 4: Comorbidities** |  |  |  |
| PACS ODD diagnosis (yes) | 3.54 | 1.36 | **.009** |
| PACS CD diagnosis (yes) | 2.32 | 1.68 | .168 |
| PACS screen anxiety/depression (yes) | -0.30 | 1.31 | .819 |
| **Class 5: Pharmacological treatment** |  |  |  |
| Cumulative intake (average daily dose*duration treatment) | 0.011 | 0.01 | **.122** |

*Note* Variables in bold are pre-selected based on a *p*-value <.15. Predictors were assessed at baseline.

**ADHD** Attention-deficit/hyperactivity disorder **CD** Conduct Disorder **CPRS-R:L** Conners’ Parent Rating Scale-Revised: Long Version **ODD** Oppositional Defiant Disorder **PACS** Parental Account of Children’s Symptoms **SDQ** Strengths and

Difficulties Questionnaire **SES** socio-economic status.

**Table 2 Supplement.** Pre-selection of Predictors for Current Overall Functioning.

|  | *b^b^* | *SE* | *p^c^* |
| --- | --- | --- | --- |
| **Class 1: Demographic variables** |  |  |  |
| Age (*yrs*) | 0.07 | 0.02 | **.001** |
| Sex (male=0, female=1) | 0.14 | 0.15 | .348 |
| SES (average educational level of the parents) | 0.03 | 0.02 | .236 |
| **Class 2: ADHD familiality** |  |  |  |
| ADHD status siblings (%of siblings with ADHD) | -0.002 | 0.002 | .538 |
| Parental ADHD status (yes, ADHD in one or both parents) | -0.30 | 0.13 | **.017** |
| **Class 3: ADHD characteristics** |  |  |  |
| CPRS-R:L symptom level (scale N) | -0.02 | 0.004 | **<.001** |
| SDQ parent-reported impairment | -0.03 | 0.01 | **.008** |
| SDQ parent-reported impairment | -0.02 | 0.01 | .295 |
| Age of onset first ADHD symptoms (*yrs*) | 0.01 | 0.03 | .679 |
| **Class 4: Comorbidities** |  |  |  |
| PACS ODD diagnosis (yes) | -0.07 | 0.11 | .530 |
| PACS CD diagnosis (yes) | -0.27 | 0.15 | **.066** |
| PACS screen anxiety/depression (yes) | -0.18 | 0.11 | **.100** |
| **Class 5: Pharmacological treatment** |  |  |  |
| Cumulative intake (average daily dose*duration treatment) | 0.00 | .0001 | **.738** |

*Note* Variables in bold are pre-selected based on a *p*-value <.15. Predictors were assessed at baseline.

**ADHD** Attention-deficit/hyperactivity disorder **CD** Conduct Disorder **CPRS-R:L** Conners’ Parent Rating Scale-Revised Long Version **ODD** Oppositional Defiant Disorder **PACS** Parental Account of Children’s Symptoms **SDQ** Strengths and

Difficulties Questionnaire **SES** socio-economic status.

**Table 3 Supplement.** Characteristics of Younger and Older Children with ADHD/C

|  | **Younger**  **<12 years** | | **Older**  **12≥years** | |
| --- | --- | --- | --- | --- |
|  | **Mean** | **SD** | **Mean** | **SD** |
| **Baseline** |  |  |  |  |
| *Demographic variables* |  |  |  |  |
| Age (*yrs*) | 9.62 | 1.70 | 14.19 | 1.52 |
| Sex *(N* / %, male) | 167 | 80.3 | 102 | 83.6 |
| SES (average educational level of the parents) | 5.16 | 2.11 | 5.73 | 2.33 |
| *ADHD familiality* |  |  |  |  |
| ADHD status siblings (% of siblings with ADHD) | 65.25 | 26.22 | 60.00 | 25.04 |
| Parental ADHD status (*N* / % ADHD in one or both parents) | 63 | 36.8 | 26 | 26.5 |
| *ADHD severity^a^* |  |  |  |  |
| CPRS-R:L Total symptom severity (scale N) | 35.92 | 8.29 | 34.82 | 9.01 |
| SDQ Impairment |  |  |  |  |
| -Parent | 12.50 | 3.81 | 11.98 | 3.95 |
| -Teacher | 8.33 | 3.10 | 7.38 | 3.31 |
| Age of onset first ADHD symptoms (*yrs*) | 2.29 | 1.15 | 2.16 | 1.51 |
| *ADHD pharmacological treatment* |  |  |  |  |
| Mean daily dose (milligram, unit equivalents) | 11.77 | 11.43 | 15.80 | 14.74 |
| Cumulative intake of psychostimulants | 47.01 | 67.62 | 63.14 | 82.00 |
| *Comorbidities* |  |  |  |  |
| PACS ODD diagnosis (yes) | 103 | 54.2 | 69 | 61.6 |
| PACS CD diagnosis (yes) | 31 | 16.3 | 23 | 20.5 |
| PACS screen anxiety/depression (yes) | 117 | 61.6 | 62 | 55.4 |
| **Follow-up** |  |  |  |  |
| *Demographic variables* |  |  |  |  |
| Age at follow-up (*yrs)* | 15.63 | 1.77 | 20.12 | 1.67 |
| *ADHD severity^a^* |  |  |  |  |
| CPRS-R:L Total symptom severity (scale N) | 23.98 | 11.17 | 22.06 | 11.68 |
| CPRS-R:L Total symptom severity change score (scale N) | 11.93 | 11.51 | 12.76 | 12.03 |
| *ADHD pharmacological treatment* |  |  |  |  |
|  |  |  |  |  |
| Mean daily dose (milligram, unit equivalents) | 23.17 | 15.67 | 20.21 | 15.76 |
| Cumulative intake of psychostimulants | 140.76 | 126.34 | 102.11 | 106.57 |
| *Status at follow-up* |  |  |  |  |
| Kiddie-Global Assessment Scale at follow-up | 6.30 | 1.18 | 6.61 | 1.03 |
| ADHD persistence (*N /* %) | 178 | 86.4 | 110 | 86.6 |
| -ADHD/C (*N /* %) | 88 | 43.8 | 51 | 43.6 |
| -ADHD/I (*N /* %) | 73 | 36.3 | 38 | 32.5 |
| -ADHD/H (*N /* %) | 13 | 6.5 | 13 | 11.1 |
| Subthreshold ADHD (*N /* %) | 17 | 8.3 | 11 | 8.7 |
| ADHD remitter (*N /* %) | 11 | 5.3 | 6 | 4.7 |
| *Comorbidities* |  |  |  |  |
| ODD (*N /* %) | 65 | 31.4 | 38 | 29.7 |
| CD (*N /* %) | 14 | 6.8 | 8 | 6.3 |
| Tic disorder (*N /* %) | 5 | 2.4 | 2 | 1.6 |
| Mood disorder (*N /* %) | 3 | 1.5 | 3 | 2.4 |
| Anxiety disorder (*N /* %) | 4 | 2.0 | 4 | 3.2 |

**ADHD** Attention-deficit/hyperactivity disorder **ADHD/C** Attention-deficit/hyperactivity disorder combined-type **ADHD/H**

Attention-deficit/hyperactivity disorder hyperactive/impulsive-type **ADHD/I** Attention-deficit/hyperactivity disorder inattentive-type **CD** Conduct Disorder **CPRS-R:L** Conners’ Parent Rating Scale-Revised: Long Version **ODD** Oppositional Defiant Disorder **PACS** Parental Account of Children’s Symptoms **SDQ** Strengths and Difficulties Questionnaire **SES** socio-economic status.

**^a^** combined measures of parent/self and teacher report.
